# Maternal High-Fat Diet Programs White and Brown Adipose Tissues In Vivo in Mice, with Different Metabolic and Microbiota Patterns in Obesity-Susceptible or Obesity-Resistant Offspring

**DOI:** 10.3390/metabo12090828

**Published:** 2022-09-02

**Authors:** Maria Angela Guzzardi, Maria Carmen Collado, Daniele Panetta, Maria Tripodi, Patricia Iozzo

**Affiliations:** 1 Institute of Clinical Physiology, National Research Council (CNR), 56124 Pisa, Italy; 2 Institute of Agrochemistry and Food Technology, National Research Council (IATA-CSIC), 46980 Valencia, Spain

**Keywords:** fetal programming, maternal high-fat diet, adipose tissue, glucose uptake, radiodensity, positron emission tomography, microbiota, adipokines

## Abstract

Maternal obesity causes metabolic dysfunction in the offspring, including dysbiosis, overeating, obesity, and type 2 diabetes. Early-life phases are fundamental for developing subcutaneous (SAT) and brown adipose tissues (BAT), handling energy excesses. Imaging of ^18^F-fluorodeoxyglucose by positron emission tomography (PET) and radiodensity by computerized tomography (CT) allows assessing adipose tissue (AT) whitening and browning in vivo and the underlying metabolic efficiency. Our aim was to examine these in vivo traits in SAT and BAT concerning gut microbiota composition in 1- and 6-month-old mice born to normal (NDoff) and high-fat diet-fed dams (HFDoff), accounting for body weight responses. We found low radiodensity (high lipids) in HFDoff SAT at 1 month, relating to an increased abundance of *Dorea* genus in the caecum and activation of the fatty acid biosynthetic pathway. Instead, low BAT radiodensity and glucose uptake were seen in adult HFDoff. Glucose was shifted in favor of BAT at 1 month and SAT at 6 months. In adults, unclassified *Enterococcaceae* and *Rikenellaceae*, and *Bacillus* genera were negatively related to BAT, whereas unclassified *Clostridiales* genera were related to SAT metabolism. Stratification of HFDoff based on weight-response, namely maternal induced obesity (MIO-HFDoff) or obesity-resistant (MIOR-HFDoff), showed sex dimorphism. Both subgroups were hyperphagic, but only obese mice had hyper-leptinemia and hyper-resistinemia, together with BAT dysfunction, whereas non-obese HFDoff had hyperglycemia and SAT hypermetabolism. In the caecum, unclassified *Rikenellaceae* (10-fold enrichment in MIO-HFDoff) and *Clostridiales* genera (4-fold deficiency in MIOR-HFDoff) were important discriminators of these two phenotypes. In conclusion, SAT whitening is an early abnormality in the offspring of HFD dams. In adult life, maternal HFD and the induced excessive food intake translates into a dimorphic phenotype involving SAT, BAT, and microbiota distinctively, reflecting maternal diet*sex interaction. This helps explain inter-individual variability in fetal programming and the higher rates of type 2 diabetes observed in adult women born to obese mothers, supporting personalized risk assessment, prevention, and treatment.

## 1. Introduction

The obesity epidemic is a global threat to health lacking effective treatment. The growing prevalence of obesity and its metabolic complications in children indicates that prevention should be started in very early life phases. Unhealthy obesity is characterized by limited subcutaneous adipose tissue (SAT) substrate storage capacity and the whitening of brown adipose tissue (BAT) at the expense of glucose and fatty acid clearance and energy consumption by, e.g., glucose oxidation and non-shivering thermogenesis [1,2,3,4].

Early life factors are fundamental in the development of AT affecting metabolic health throughout the course of life. Among them, maternal overweight during gestation and lactation, mostly due to a high-fat diet (HFD), has become very common. In rodents, it impairs fetal brown adipogenesis and myogenesis and promotes white adiposity and metabolic complications [5,6]. In humans, maternal obesity and a high caloric dietary pattern predict obesity and type 2 diabetes in the offspring [7,8]. Accredited mechanisms include the transfer of maternal circulating or breastmilk glucose and free fatty acids to the fetus and neonate, promoting hyperinsulinemia and adipogenesis [9]. In addition, the influence of maternal diet on gut microbiota development in the offspring is an emerging modifiable factor, potentially controlling adiposity and energy metabolism from early life.

The number of studies addressing the gut microbiota-SAT/BAT axis in maternal obesity is limited but supportive. In humans, maternal HFD or obesity seems to influence fetal and children’s microbiota [10,11]. Children born to mothers taking antibiotics during gestation have higher BMI, fat mass, and waist circumference than children of untreated mothers [12,13,14]. Notably, the impact of early life antibiotics on adiposity remains after treatment, even though the microbiota recovers, highlighting that AT alterations in the critical time window can have long-term effects [15]. Breastmilk composition is also affected by maternal weight and has been postulated to modify AT development via gut microbiota [16,17,18,19].

Large gaps in knowledge encompass the effects of maternal HFD on AT browning/whitening from early to adult life and the identification of the gut bacteria involved. Sexual dimorphism was shown to affect BAT whitening in male mice born to HFD dams and AT transcriptomics, but in that study, all groups were exposed to post-natal HFD [20]. Moreover, the current knowledge is built on measurements of AT mass or ex vivo gene expression, lacking translation into functional in vivo effects, namely tissue-specific substrate metabolism, underlying storage capacity, and thermogenesis. Metabolic imaging by positron emission, computerized tomography (PET-CT), is the gold standard for the simultaneous in vivo quantification of AT glucose uptake (GU) and whitening/browning. High versus low CT radiodensity and GU have been validated to reflect histological and molecular evidence of AT browning (small multilocular cytoplasmic lipid droplets with mitochondria richness and respective gene expression) versus whitening (larger unilocular lipid depots with lipogenic gene profiles) [21,22,23,24,25,26].

The novelty and aim of this study was to examine in vivo GU and radiodensity in SAT and BAT concerning gut microbiota in young and adult mice born to normal and HFD dams. Individual responses to maternal HFD were investigated to dissect the direct from the indirect (post-natal obesity mediated) effects in adults. We expected progressive whitening of BAT and SAT and impairment in GU from early age to adulthood, and conjectured that a restricted panel of bacteria could partially explain age-specific AT functional characteristics.

## 2. Results

### 2.1. Metabolic Characteristics

**Figure 1:**
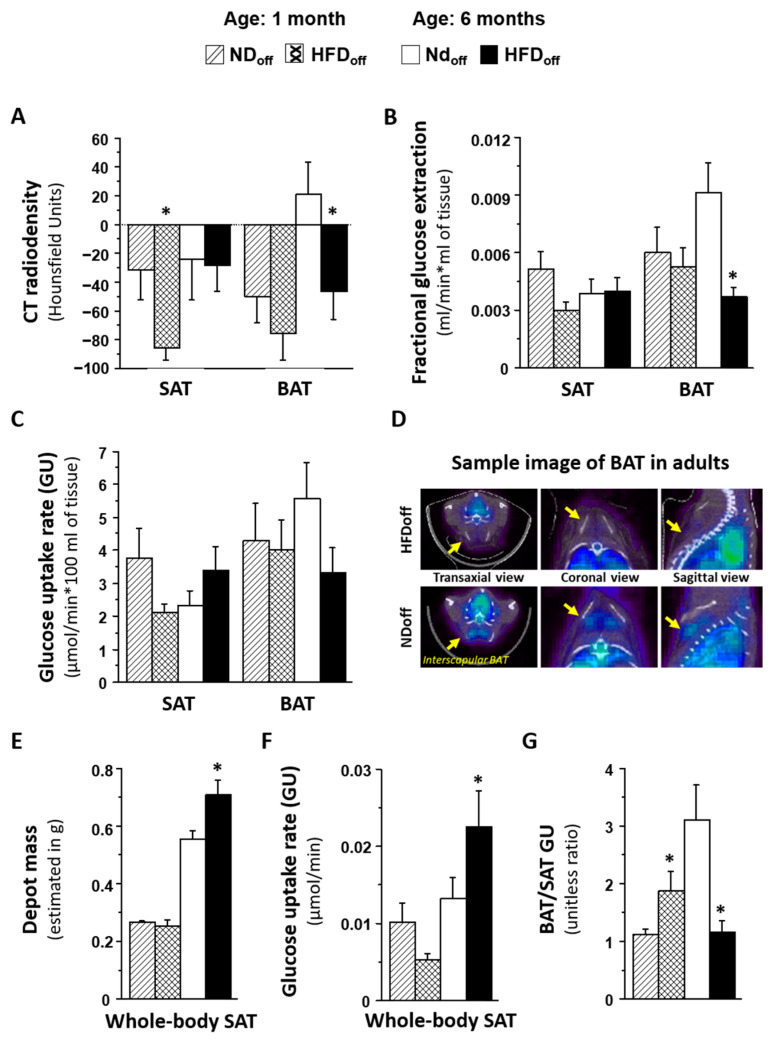
Phenotype of mice born to HFD or ND dams, including BAT and SAT lipid content (**A**), given by negative values in CT radiodensity, glucose metabolism (**B**–**D**) by PET imaging, features of whole-body SAT (**E**,**F**), and the metabolic balance between BAT and SAT (**G**), in early and adult life. The sample images in D shows interscapular BAT ^18^FDG levels (Bq/mL) in transaxial, coronal, and sagittal views, in two representative cases (HFDoff: fractional extraction rate constant = 0.003 mL/min, GU = 2.4 µmol/min * 100 mL; NDoff: fractional extraction rate constant = 0.013 mL/min, GU = 9.4 µmol/min * 100 mL). * *p* < 0.05 vs. age-matched NDoff group. NDoff = offspring born to ND dams; HFDoff = offspring born to HFD dams.

In 1-month-old offspring born to HFD dams (HFDoff) (Table 1), SAT was characterized by a significant three-fold reduction in radiodensity and a tendency towards low GU as compared to offspring born to ND dams (NDoff). BAT did not show any group difference. Thus, the BAT/SAT metabolic rate ratio increased by 70% in HFDoff, suggesting a relative imbalance in GU in favor of BAT at this young age. In 6-month-old HFDoff (Figure 1), radiodensity and fractional glucose extraction were severely reduced in BAT compared to age-matched NDoff. Consequently, the BAT/SAT metabolic rate ratio was impaired by 70% in adult HFDoff. The estimated mass of whole-body SAT was 28% greater in HFDoff (*p* = 0.0008), leading to a significant two-fold elevation in whole-depot GU compared to NDoff (*p* = 0.038).

**Figure 2:**
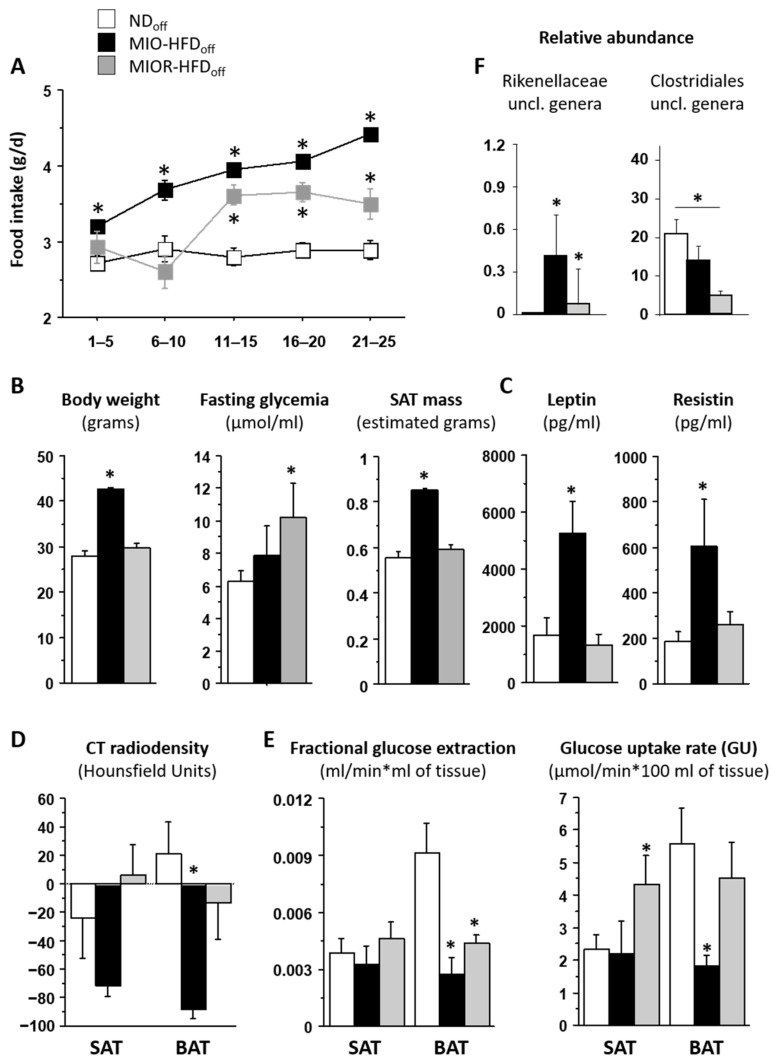
Phenotype of mice born to HFD or ND dams. The group of offspring born to HFD dams (HFDoff) is stratified according to the presence or absence of adulthood obesity (MIO, MIOR). Food intake, body weight, and metabolic and adipokine profiles are shown in (**A**–**C**); BAT and SAT lipid content and glucose metabolism are given in (**D**,**E**), and microbiota signatures are displayed in (**F**). * *p* < 0.05 vs. age-matched NDoff group. MIO-HFDoff = offspring born to HFD dams with maternally induced obesity; MIOR-HFDoff = offspring born to HFD dams but resistant to maternally induced obesity; uncl. = unclassified.

Considering that interindividual health responses to maternal HFD can vary, and to dissect the direct from the indirect (post-natal obesity mediated) effects on adult HFDoff, we separated mice that developed maternally induced obesity (MIO-HFDoff, *n* = 4, body weight 42.8 ± 0.3 g, *p* < 0.0001 vs. NDoff) from those who were more resistant (MIOR-HFDoff, *n* = 5), i.e., those whose adulthood body weight fell in the normal range (29.6 ± 1.1 vs. 27.8 ± 1.5 g, n.s. vs. NDoff, *p* < 0.0001 vs. MIO-HFDoff). This revealed that only MIO-HFDoff were characterized by significantly low BAT radiodensity and low BAT GU. In contrast, MIOR-HFDoff had normal BAT values compared to ND but higher SAT GU (Figure 2). Leptin and resistin were also elevated only in MIO-HFDoff compared to MIOR-HFDoff and NDoff. Conversely, hyperglycemia was present only in MIOR-HFDoff and not in MIO-HFDoff. Considering that the MIO-HFDoff phenotype seemed related to the male sex, we repeated the analysis in sex*maternal subgroups, obtaining similar results (Table 1), i.e., maternal HFD affected male and female offspring differently, inducing obesity and low BAT GU in males, and hyperglycemia and high SAT GU in females. In addition, hypertriglyceridemia was associated with the male sex in adults, whereas hyperglycemia, low body weight, and SAT mass were already seen in females at weaning. In 1-month old offspring, SAT radiodensity was negatively related to plasma resistin levels (r = −0.56, *p* = 0.020). In 6 months-old mice, BAT and SAT radiodensities and BAT glucose extraction rates were negatively related to circulating resistin, leptin and/or triglyceride levels (BAT radiodensity vs. resistin r = −0.50, *p* = 0.03, leptin r = −0.68, *p* = 0.001, triglycerides r = −0.49, *p* = 0.052; SAT radiodensity vs. resistin r = −0.54, *p* = 0.020, leptin r = −0.72, *p* = 0.0007; BAT glucose extraction vs. resistin r = −0.48, *p* = 0.038, triglycerides r = −0.48, *p* = 0.059).

**Table 1:** Metabolic profile and imaging data stratified by offspring sex, showing similarities of MIO vs. male and MIOR vs. female groups, as explained in the legend of Figure 3.

	1 Month Old		6 Months Old	
**Female**	**ND_off_**	**HFD_off_**	**ND_off_**	**HFD_off_**
Body weight (g)	17 ± 1	**13 ± 1 ^**	26 ± 2	27 ± 0
SAT mass (g)	0.26 ± 0.02	**0.19 ± 0.01 ^**	0.51 ± 0.04	0.54 ± 0.00
F-Glycemia (mmol/L)	6.3 ± 0.2	8.5 ± 1.2 *	5.3 ± 0.3	10.5 ± 0.3 *
Scan glycemia (mmol/L)	4.6 ± 0.6	6.7 ± 1.2	5.6 ± 0.9	14.5 ± 3.2 *
Triglycerides (mmol/L)	0.794 ± 0.004	0.805 ± 0.015	0.790 ± 0.000	1.123 ± 0.333
SAT CT (HU)	−34 ± 48	−94 ± 8	−2.2 ± 31	−19 ± 3
BAT CT (HU)	−56 ± 22	−78 ± 31	61 ± 20	−22 ± 43 ^
SAT GU (µmol/min*100 g)	1.9 ± 0.7	1.7 ± 0.3	2.1 ± 0.3	5.1 ± 2.0 *
Whole SAT GU (µmol/min)	0.005 ± 0.002	0.003 ± 0.002	0.011 ± 0.002	0.027 ± 0.011 *
BAT GU (µmol/min*100 g)	2.0 ± 1.0	3.5 ± 0.8	4.9 ± 1.0	6.4 ± 2.2
BAT/SAT GU	1.0 ± 1.8	2.1 ± 0.4	2.6 ± 0.3	1.3 ± 0.1 *
**Male**	**ND_off_**	**HFD_off_**	**ND_off_**	**HFD_off_**
Body weight (g)	18 ± 0	19 ± 2	31 ± 1	38 ± 2 *
SAT mass (g)	0.27 ± 0.01	0.29 ± 0.02	0.63 ± 0.02	0.76 ± 0.05 *
F-glycemia (mmol(L)	8.3 ± 0.6	7.8 ± 0.4	6.4 ± 0.7	7.2 ± 0.8
Scan glycemia (mmol/L)	7.7 ± 0.9	8.1 ± 0.7	7.2 ± 1.0	7.7 ± 1.1
Triglycerides (mmol/L)	0.819 ± 0.028	0.931 ± 0.141	0.790 ± 0.000	1.155 ± 0.101 *
SAT CT (HU)	−30 ± 21	−81 ± 13	−51 ± 53	−31 ± 23
BAT CT (HU)	−46 ± 28	−74 ± 26	−39 ± 27	−53 ± 23
SAT GU (µmol/min*100 g)	5.0 ± 1.3	2.3 ± 0.4 ^	2.5 ± 1.0	2.9 ± 0.8
Whole SAT GU (µmol/min)	0.014 ± 0.004	0.007 ± 0.001	0.015 ± 0.006	0.021 ± 0.005
BAT GU (µmol/min*100 g)	5.8 ± 1.5	4.3 ± 1.4	6.6 ± 2.4	2.4 ± 0.5 *
BAT/SAT GU	1.2 ± 0.1	1.8 ± 0.5	3.8 ± 1.3	1.1 ± 0.3 *

* ANOVA; ^ *T*-test. Values are means ± SEM. F = fasting; HU = radiodensity in Hounsfield Units * *p* < 0.05, ^ *p* = 0.06 vs. age-matched NDoff.

### 2.2. Relationship with Microbiota and Metabolic Pathways

Univariate and FDR-adjusted associations between imaging parameters and microbiota composition or KEGG-derived metabolic pathways were examined (Table 2 and Table 3). Most correlations were found in the caecum, linking AT glucose extraction and GU with taxa belonging to *Bacilli*, *Clostridia* (Firmicutes phylum), or *Bacteroidia* (Bacteroidetes phylum) classes.

At 1 month of age, the relative abundance of the *Dorea* genus in the caecum was strongly and inversely related to SAT fractional glucose extraction; a lower SAT radiodensity at 1 month was predicted by higher fatty acid biosynthesis pathway in the caecum microbiota. In adults, at the genus level, unclassified *Enterococcaceae* and *Bacillus* genera were negatively related to BAT GU and/or glucose extraction, and unclassified *Rikenellaceae* and *Bacillus* genera were negative, whereas unclassified *Clostridiales* genera were positively related to the BAT/SAT GU ratio. Whole-body SAT GU was negatively related to *rc44*, unclassified members of *Christensenellaceae*, *Ruminococcaceae*, *Erysipelotrichaceae*, and *Clostridiales*, as well as to *Coprococcus*, *Anaerotruncus*, *Oscillospira*, and *Dehalobacteriumgenera*. The caecum bacterial secretion system pathway was negatively related to the BAT/SAT GU ratio and positively associated with whole-body SAT GU in adults. Among the above caecum bacteria, we observed a 10-fold enrichment in unclassified *Rikenellaceae* genera in MIO-HFDoff, and a 4-fold deficiency in unclassified *Clostridiales* in MIOR-HFDoff (Figure 2F).

## 3. Discussion

Novelties of this study were characterizing in vivo functional SAT and BAT metabolism and investigating their relationships with the gut microbiota in young and adult offspring of HFD mothers. In particular, weaning is the period of life with the most intensive microbiota transformation, whereas the adult stage is the period manifesting health consequences of maternal obesity. Notably, not all mice react to maternal obesity similarly, and we observed important differences between offspring developing or not developing obesity as a consequence of maternal HFD*sex interaction.

The first important finding of this study concerns the time course of AT abnormalities seen in the offspring of HFD dams. Our results indicate that SAT is first affected, and undergo marked whitening, providing the in vivo counterproof of the histological evidence of adipocyte hypertrophy in another study [27]. These early abnormalities were dependent on fat exposure in utero and during lactation through maternal HFD. They were not dependent on body weight or the metabolic profile, subsequent to this and the previous study [27]. Our findings suggest that the gut microbiota may be one mediator of these early SAT findings. The higher relative abundance of the *Dorea* genus (Firmicutes phylum, *Clostridia* class) and activation of the microbiota fatty acid biosynthetic pathway (FABP) in the caecum were predictors of the low SAT glucose extraction (*Dorea*) and low radiodensity (FABP) observed in HFDoff at 1 month. This causal interpretation is supported by the notion that HFD exposure increases *Dorea* genus abundance in mice, and this genus is a confirmed biomarker of human adiposity [28,29,30]. In addition, the activation of the fatty acid synthetic pathway establishes a coherent mechanistic link with low radiodensity, which reflects lipid storage and adipocyte size in AT. In turn, SAT radiodensity was inversely predictive of circulating resistin levels already at 1 month, which may imprint the subsequent increase in adult HFDoff, implying early seeding of persistent SAT endocrine dysfunction. The 70% elevation in the BAT/SAT ratio indicates that the relative proportion of glucose flowing into ATs is shifted towards BAT in HFDoff. This may contribute to maintaining normal fasting substrate levels in this early life phase. Previous ex vivo tissue analyses of BAT have shown an increase in triglyceride content, white AT markers, and reduced mitochondriogenesis in fetuses born to HFD dams [5]. In vivo, we found a mild tendency towards BAT whitening at weaning, and a significant decline in BAT radiodensity and glucose extraction in adult HFDoff. The loss of metabolic BAT activity in our adult HFDoff is consistent with the evidence of reduced thermogenesis in adult mice born to obese dams [16]. Altogether, this suggests that BAT at 1 month of age reacts to compensate for SAT dysmetabolism, and the progressive overweight starting after weaning until adulthood results in a fully dysfunctional BAT and severe suppression of the BAT/SAT GU ratio, with a two-fold elevation in whole-depot SAT GU. The latter is the product of tissue glucose extraction, plasma glucose levels, and whole-body SAT mass, thereby summing up the contribution of the main health consequences of maternal HFD, namely adiposity and high glycemia.

To distinguish the role of maternally induced obesity from non-obesity-mediated effects, we stratified HFDoff based on their weight response to maternal HFD, noting that approximately half of the offspring became frankly obese (MIO-HFDoff), with the remaining half falling in the NDoff weight range (MIOR-HFDoff). The salient finding was that only obese mice showed a five-fold reduction in BAT radiodensity and a three-fold decline in BAT GU, together with an increase in resistin levels. In contrast, these defects were not seen in MIOR-HFDoff. Instead, this group showed an elevation in SAT GU, which was driven by hyperglycemia, whereas MIO-HFDoff were normoglycemic. We further discovered that MIO-HFDoff were males, and that similar dimorphisms in BAT, SAT, body weight, and glycemic responses were seen in gender comparisons. These observations demonstrate that maternal HFD impairs SAT metabolism and glucose tolerance in females, independent of offspring obesity. Instead, the impacts on BAT depend on maternally induced obesity in males and confer some degree of protection against hyperglycemia. Food intake was highest in MIO-HFDoff, and was significantly higher in MIOR-HFDoff compared to NDoff from 3 to 6 months of age. High food intake, with normal BAT metabolism, normal body weight, and high SAT radiodensity in MIOR-HFDoff, suggests a limited AT capacity to store energy excesses, which has been related to insulin resistance and deficient glucose-sinking efficacy in humans [2].

BAT whitening and low SAT/VAT mass in male offspring with gender-dependent AT transcriptomics were observed in mice exposed to HFD during uterine and post-natal life until adulthood [20]. In that study, male offspring exposed to HFD post-weaning (whether born to HFD or ND mothers) developed hyperglycemia. Female offspring seemed metabolically protected, which is at variance with human studies showing that women born to obese mothers are more prone than men to develop type 2 diabetes during adult life [8]. Our study extends this previous knowledge, showing that hyperglycemia occurs only in females born to HFD mothers if a normal diet is administered after weaning. This suggests a gender-diversified response to the match [20] or mismatch between in utero and post-weaning diets, in which a match of HFD results in detrimental outcomes in males, whereas females compensate with SAT expansion [20]. Conversely, mismatch (HFD before and normal diet after weaning in our current study) prevents SAT storage capacity in females and generates glucose overloading.

Food intake is an energy source and an important modulator of the gut microbiota. Notably, BAT outcomes were significantly related to microbiota composition in adults. More specifically, very strong (FDR-adjusted) associations were found between BAT dysmetabolism and relative abundance of *Bacillus*, Unclassified *Enterococcaceae*, Unclassified *Rikenellaceae* (negative), and Unclassified *Clostridiales* genera (positive) in the caecum. We have previously shown that caecum overabundance of Unclassified *Rikenellaceae* and depletion of Unclassified *Clostridiales* genera were biomarkers of adult mice born to HFD dams. Likewise, the elevation in whole-body SAT GU observed in adult HFDoff was tightly related (FDR-adjusted) with the depletion in *rc44*, *Anaerotruncus*, and Unclassified *Clostridiales* genera, which were hallmarks of adult mice born to HFD dams. So, these bacterial profiles connect maternal HFD with AT outcomes in adult mice. One important observation was that maternal HFD induced a different pattern in these bacteria in obesity-prone MIO-HFDoff and obesity-resistant hyperglycemic MIOR-HFDoff. Compared to NDoff, the abundance of Unclassified *Rikenellaceae* bacteria was 10 folds higher in MIO-HFDoff and normal in MIOR-HFDoff, whereas MIOR-HFDoff showed a 4-fold reduction in Unclassified *Clostridiales* levels. Notably, treatment with *Clostridiaceae* was shown to directly up-regulate lipid transport and synthesis genes both in vivo and in vitro, suggesting that the deficiency of these bacteria was involved in the inability of (food-derived) energy excess storage seen in ATs in our MIOR-HFDoff [31]. In KEGG pathway analysis, only the bacteria secretion system was associated with BAT and SAT outcomes after FDR adjustment in our study. Interestingly, this system regulates nutrient acquisition and is overexpressed in relation to metabolic diseases, such as type 2 diabetes [32].

The main limitation of the study is the limited sample size of HFDoff subgrouping, but we felt it is important to show the remarkable differences observed between MIO vs. MIOR subgroups, contributing to explaining the inter-individually diverse response to early-life exposures to maternal HFD and the gut microbiota. Moreover, the maternal sample was not powered to establish if the proneness towards MIO vs. MIOR phenotypes may prevail in mothers with given characteristics, though we noted that in some cases the same mother gave birth to both phenotypes. This remains an important question to be addressed in future studies. Our data do not provide a mechanistic explanation for the evidence that females and males were more prone to develop one or the other phenotype; gender dimorphic health responses are commonly also noted in humans born to obese mothers [8], but the underlying cause remains to be established. Our data implicate a possible effect of the microbiota to hamper SAT expansion, resulting in hyperglycemia, as we know that the microbiota is sex-related [33].

**Figure 3:**
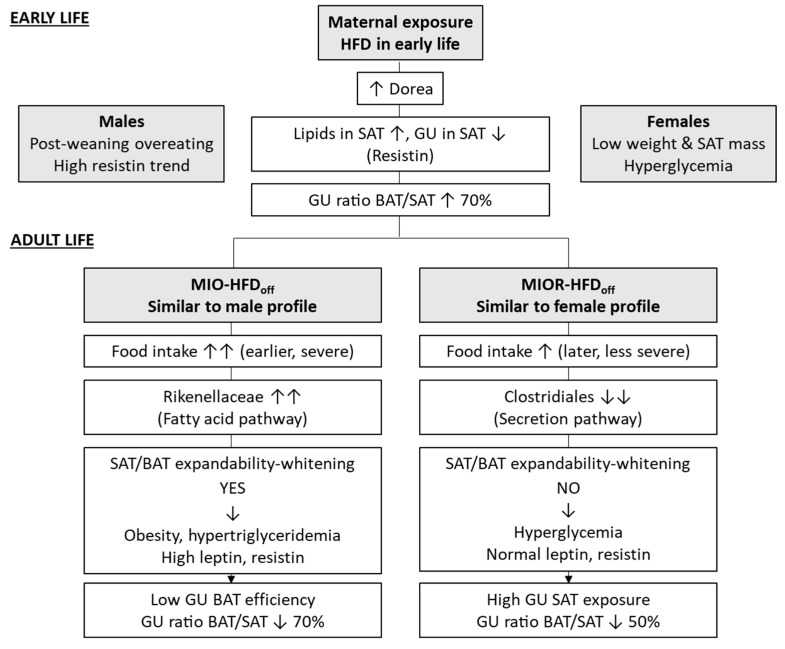
Diagram of features underlying the distinct phenotypes in early- and adult-life, as both induced by maternal HFD exposure during pregnancy and lactation. The top section refers to the weaning age of the offspring when obesity or hyperglycemia have not developed, though differences between sexes suggest early phenotypic seeding. The bottom section highlights distinct patterns of fat and microbiota development that relate to different complications in later life, consistent with the human situation, in which maternal obesity leads to greater odds-ratios of obesity or diabetes [8], but many offspring remain normal weight or non-diabetic. Sex was analyzed to point out that a majority of individuals developing obesity in response to maternal HFD were males and the ones developing hyperglycemia were mostly females. In humans, we know that women born to obese mothers are more prone than men to develop diabetes in later life [8]. Though obesity/diabetes proneness and sex seem to overlap largely in our results, the two analyses are conceptually independent and shown separately until mechanisms are clarified.

We conclude that early life HFD exposure causes SAT whitening, reducing substrate utilization, redirecting glucose towards BAT, and the overabundance of the caecum *Dorea* genus may mediate these findings. BAT whitening and dysmetabolism become the major targets of maternal HFD in later life, shifting substrates towards SAT and promoting SAT expansion. One important novel finding was that maternal HFD, leading to greater food intake in adult offspring, resulted in a dichotomic response, translating into either (1) a lean-hyperglycemic phenotype, characterized by *Clostridiales* members depletion, SAT glucose overexposure with limited SAT and BAT storage capacity, prevalent in females, or (2) an obese-normoglycemic phenotype, with *Rikenellaceae* enrichment, SAT expansion, and BAT whitening, prevailing in males. From a clinical standpoint, once verified in humans, our results (a) highlight the importance of monitoring the offspring of HFD mothers, even if (or especially if) the offspring do not develop obesity, given the potential risk of adulthood diabetes, (b) show that proneness to obesity or diabetes overlaps with sex, though the two associations are conceptually distinct, and were kept as separate, and (c) suggest the possibility of microbiota-based prevention to be proven in ad-hoc cause-effect studies. Our data interpretation is recapitulated in Figure 3. Via the above dysfunctional loops, both ATs contribute interactively to setting the stage for adulthood metabolic disease and explaining the frequently reported inter-individual variability and the risk of type 2 diabetes in women born to obese mothers. Capturing the interaction between food intake, gut microbiota, and metabolic responses may support risk-assessment and personalized treatment.

## 4. Materials and Methods

### 4.1. Animal Model and Study Design

The study design and methods have been previously described [34]. In brief, we studied 38 offspring born to B6129SF2/J dams (stock no:101045, The Jackson Laboratory, Bar Harbor, Maine), exposed to a normal diet (ND 11% kcals from fat, *n* = 5), or a high-fat diet (HFD 58% kcals from fat, *n* = 4) for 3 months before mating, through gestation and lactation. After weaning, offspring were fed ND.

Animals were housed under standard conditions (22 °C, 12-h light/dark cycles), with ad libitum access to food and water. Offspring were imaged with PET-CT at the time of weaning (1 month of age, *n* = 19, NDoff/HFDoff *n* = 11/8) or adulthood (6 months of age, *n* = 19, NDoff/HFDoff *n* = 10/9).

After imaging, offspring were euthanized by anesthetic overdose. Blood samples were collected to determine circulating triglyceride and adipokine levels. Also, gut contents from the colon and caecum were collected to measure microbiota composition, as previously described [34,35].

### 4.2. PET-CT Imaging

*Acquisition*. Imaging of 18FDG was performed under fasting conditions (PET-CT IRIS, Inviscan SAS, Strasbourg, France) and isofluorane anaesthesia (IsoFlo^®^, Abbott Laboratories, IL, USA), as previously described [35,36]. Briefly, after CT scanning, 18FDG was injected intraperitoneally, followed by a 60-min whole-body dynamic PET scan, and glycemia was measured in tail blood by a glucometer (OneTouch, Johnson&Johnson Medical SpA, Pomezia, Italy).

*Processing.* PET data were corrected for dead time, random coincidences, and radioactive decay and reconstructed by a 3D-Ordered Subset Expectation Maximization (3D-OSEM) algorithm. CT images were corrected for beam hardening and ring artifacts, reconstructed with cone-beam filtered back-projection (FBP), and calibrated in Hounsfield units (HU). PET and CT images were fused, and regions of interest were manually drawn on PET-CT images in correspondence of lower abdominal SAT and interscapular BAT using the AMIDE Medical Image Data Examiner 1.0.4 (http://amide.sourceforge.net/, accessed on 10 July 2022). Tissue radiodensity HU and tissue time activity curves were obtained from CT and PET images, respectively. The steady-phase of these curves was normalized to the integrated blood activity from injection to steady-phase, representing the fractional glucose extraction (GE), and multiplied by glycemia to obtain GU [35]. Then, GU per unit of tissue mass was multiplied by the whole mass of SAT by using published CT-based estimations of 1.5% (young mice) and 2.0% (adult mice) of body weight, as validated by others [37].

### 4.3. Biochemical Analyses

Blood samples were collected at the end of the imaging procedures to determine circulating markers levels. Triglyceride levels were determined by a bench clinical chemistry analyzer (Reflovet^®^ Plus, a scil animal care company S.r.l., Treviglio, Italy). Leptin and resistin levels were measured by Luminex^®^ xMAP^®^ technology (Merck-Millipore Corp., Boston, MA, USA). They have been previously reported [34,36] and were only used here to examine associations with AT metabolism.

### 4.4. Gut Bacteria 16SrRNA Gene Sequencing

*Sample processing*. The microbiome was analyzed in ceacum and colon content, as previously reported [34,36]. Briefly, after DNA purification (MasterPure Complete DNA&RNA Purification Kit (Epicentre, Illumina, San Diego, WI, USA), and normalization to 10 ng/μL (Qubit^®^ 2.0 Fluorometer, Life Technology, Carlsbad, CA, USA), the V3–V4 region of the 16S rRNA gene was amplified by PCR using Illumina adapter overhang nucleotide sequences according to Illumina protocols. The multiplexing step was performed using a Nextera XT Index Kit (Illumina, San Diego, CA, USA). A Bioanalyzer DNA 1000 chip (Agilent Technologies, Santa Clara, CA, USA) was used to check the PCR product. Libraries were sequenced using a 2 × 300 bp paired-end run (MiSeq Reagent kit v3) on a MiSeq-Illumina platform (FISABIO sequencing service, Valencia, Spain) according to the manufacturer’s instructions (Illumina). R1 and R2 from sequencing were joined using fastq-join from the ea-tools suite (http://code.google.com/p/ea-utils, accessed on 11 July 2022).

*Data analysis*. Data were obtained using an ad-hoc pipeline written in an R Statistics environment, and data processing was performed by a QIIME pipeline (version 1.9.0). Chimeric sequences and sequences that could not be aligned were removed. The clustered sequences were utilized to construct OTUs tables (97% identity), the taxonomical assignment was based on the Greengenes database v13.8, and microbial relative abundances with Total Sum Scaling (TSS) normalization at phylum, family, and genus levels were obtained. Sequences not taxonomically classified or belonging to cyanobacteria and chloroplasts (representing ingested plant material) were removed. The microbiota functionality was predicted using the PICRUSt 1.0.0, and the Kyoto Encyclopedia of Genes and Genomes (KEGG) was used to explore microbiota function [38].

### 4.5. Statistical Analyses

Results are presented as mean ± sem. Metabolic and imaging data between age-matched groups were compared by analysis of variance, and standard regression analyses were used to evaluate associations. Wilcoxon-rank test was used to compare specific microbial taxa between groups. Spearman’s Rho correlation analysis was used to explore univariate associations between bacteria taxa relative abundances and imaging parameters. FDR correction was applied in multiple comparisons/associations analyses, and *p* values ≤ 0.05 were considered statistically significant.

## Figures and Tables

**Table 2:** Relationships linking imaging data and microbiota.

Parameter	Level	Bacteria Taxa	R	*p*	*p* FDR-Corr	Mean Abund
CAECUM: Age 1 month						
**SAT GE**	Genus	Dorea	−0.7	0.0006	0.023	1.01
CAECUM: Age 6 months						
**BAT GE**	Order	Bacillales	−0.67	0.0024	0.038	2.97
	Family	Enterococcaceae	−0.78	0.0001	0.005	1.55
	Family	Bacillaceae	−0.72	0.0008	0.013	1.53
	Family	Streptococcaceae	−0.63	0.0054	0.042	0.03
	Family	Peptostreptococcaceae	0.62	0.0061	0.042	2.02
	Family	Aerococcaceae	−0.62	0.0066	0.042	0.42
	Genus	Unclassified.Enterococcaceae	−0.79	0.0001	0.004	1.54
	Genus	Bacillus	−0.72	0.0008	0.020	1.53
**BAT GU**	Family	Enterococcaceae	−0.77	0.0002	0.006	1.55
	Family	Streptococcaceae	−0.66	0.0027	0.041	0.03
	Family	Staphylococcaceae	−0.64	0.0039	0.041	0.98
	Genus	Unclassified.Enterococcaceae	−0.79	0.0001	0.004	1.54
**BAT/SAT GU**	Phylum	Proteobacteria	−0.67	0.0034	0.024	3.42
	Family	Rikenellaceae	−0.73	0.0009	0.014	0.20
	Family	Unclassified.Clostridiales	0.71	0.0014	0.015	16.01
	Genus	Bacillus	−0.79	0.0002	0.009	1.53
	Genus	Unclassified.Rikenellaceae	−0.73	0.0009	0.021	0.20
	Genus	Unclassified.Clostridiales	0.71	0.0014	0.022	16.01
**Whole SAT GU**	Family	Ruminococcaceae	−0.8	0.0001	0.004	6.67
	Family	Peptococcaceae	−0.72	0.0011	0.013	0.38
	Family	Christensenellaceae	−0.72	0.0012	0.013	0.14
	Family	Unclassified.Clostridiales	−0.69	0.0021	0.017	16.01
	Family	Dehalobacteriaceae	−0.64	0.0054	0.035	0.07
	Genus	rc44	−0.72	0.0011	0.017	0.38
	Genus	Unclassified.Christensenellaceae	−0.72	0.0012	0.017	0.14
	Genus	Unclassified.Ruminococcaceae	−0.71	0.0013	0.017	3.85
	Genus	Coprococcus	−0.71	0.0015	0.017	0.62
	Genus	Anaerotruncus	−0.7	0.0018	0.017	0.02
	Genus	Unclassified.Clostridiales	−0.69	0.0021	0.017	16.01
	Genus	Oscillospira	−0.65	0.0044	0.030	2.01
	Genus	Dehalobacterium	−0.64	0.0054	0.032	0.07
	Genus	Unclassified.Erysipelotrichaceae	−0.61	0.0093	0.050	1.14

Spearman rank correlations. GE: fractional glucose extraction; GU: glucose uptake.

**Table 3:** Relationships linking imaging data and metabolic pathways.

Parameter	Pathway	R	*p*	*p* FDR-corr
CAECUM: Age 1 month				
**SAT CT**	Inositol.phosphate.metabolism	0.84	1.59 × 10^−5^	0.003
	Replication.recombination.and.repair.proteins	−0.78	1.49 × 10^−4^	0.009
	Tetracycline.biosynthesis	−0.77	1.74 × 10^−4^	0.009
	Fatty.acid.biosynthesis	−0.77	1.75 × 10^−4^	0.009
	Chromosome	−0.71	1.06 × 10^−3^	0.045
**SAT GE**	Nitrotoluene.degradation	−0.82	3.42 × 10^−5^	0.007
CAECUM: Age 6 months				
**BAT/SAT GU**	Bacterial.secretion.system	−0.8	0.0001	0.024
**Whole SAT GU**	beta.Lactam.resistance	0.78	0.0002	0.023
	Flagellar.assembly	−0.77	0.0003	0.023
	Transcription.related.proteins	0.77	0.0003	0.023
	Bacterial.chemotaxis	−0.75	0.0005	0.026
	Bacterial.motility.proteins	−0.74	0.0006	0.026
	Chaperones.and.folding.catalysts	0.74	0.0007	0.026
	Sulfur.relay.system	0.71	0.0013	0.039
	Flavone.and.flavonol.biosynthesis	−0.70	0.0018	0.048
	Bacterial.secretion.system	0.69	0.0021	0.050

Spearman rank correlations. GE: fractional glucose extraction; GU: glucose uptake. The table shows FDR-significant metabolic pathways in cecum microbiota, subtending the phenotypic parameters in the first column. The most straightforward pathways in the context of AT and glucose metabolism are commented on in the text, e.g., higher fatty acid biosynthesis is consistent with a lower CT radiodensity, indicative of greater lipid content in SAT, and the bacterial secretion system is involved in nutrition and metabolic disorders, and here relates positively with SAT and negatively with BAT metabolism. The sulfur relay system is also often connected to metabolic features. The other pathways showing significant correlations are less straightforwardly interpretable but are provided for completeness and hypothesis generation.

## Data Availability

The data presented in this study are available on request from the corresponding author, as they have not yet been uploaded to a public database.
